# Establishing a core domain set for early-phase clinical trials of electrical stimulation interventions for tinnitus in adults: an online Delphi study

**DOI:** 10.1136/bmjopen-2023-079769

**Published:** 2025-03-04

**Authors:** Bas Labree, Derek Hoare, Kathryn Fackrell, Deborah A Hall, Lauren E Gascoyne, Magdalena Sereda

**Affiliations:** 1 NIHR Nottingham Biomedical Research Centre, Nottingham, UK; 2 Hearing Sciences, Mental Health and Clinical Neurosciences, School of Medicine, University of Nottingham, Southampton, UK; 3 Wessex institute, University of Southampton, Southampton, UK; 4 Department of Psychology, School of Social Sciences, Heriot-Watt University, Edinburgh, UK; 5 Sir Peter Mansfield Imaging Centre, University of Nottingham, Nottingham, UK

**Keywords:** Patient Reported Outcome Measures, Electric Stimulation Therapy, Internet, Otolaryngology

## Abstract

**Abstract:**

Tinnitus is the awareness of sound in the ear or head in the absence of an external source. It affects around 10%–15% of people, and current treatment options are limited. Experimental treatments include various forms of electrical stimulation of the brain. Currently, there is no consensus on the outcomes that should be measured when investigating the efficacy of this type of intervention for tinnitus.

**Objective:**

This study sought to address this issue by establishing a core domain set: a common standard of what specific tinnitus-related complaints are critically important to assess in all clinical trials of electrical stimulation-based interventions for tinnitus.

**Design:**

A two-round Delphi survey was conducted, followed by a stakeholder consensus meeting to identify a core domain set.

**Setting**

All data collection took place online

**Participants:**

Participants represented one of two stakeholder groups: patients with lived experience of tinnitus and professionals with relevant clinical, commercial or research experience.

**Results:**

Stakeholders achieved consensus on the inclusion of ability to ignore, concentration, treatment satisfaction, helplessness (lack of control) and tinnitus intrusiveness in the core domain set, in addition to adverse effects.

**Conclusion:**

This study established a core domain set for the evaluation of electrical stimulation-based interventions for tinnitus via an e-Delphi study. This core domain set will act as a minimum standard for reporting in future clinical trials of electrical stimulation interventions for tinnitus. Standardisation will facilitate comparability of research findings.

**STRENGTHS AND LIMITATIONS OF THIS STUDY**

- This study successfully established a core outcome domain set for electrical stimulation-based interventions for tinnitus, ensuring greater consistency across future trials and facilitating meta-analysis of the results of such trials.
- Online data collection, in particular during the consensus meeting, may have been a barrier to participation by patients with hearing loss.
- This core outcome set is sufficiently intervention-specific while reflecting the priorities of people with tinnitus as well as the wider clinical and research community.

## Introduction

 Tinnitus is the sensation of noise, ringing, buzzing or hissing sounds perceived in the ears or head.^1^ In most cases, tinnitus is only perceived subjectively. Approximately 65 million people in Europe^2^ and more than 50 million people in the USA experience tinnitus. Tinnitus can be chronic and disabling and is associated with a diverse range of complaints, including perceived loudness, sleep problems, difficulties in listening and concentration, and effects on psychological well-being, daily life and general health.^3^,^6^ Tinnitus may also negatively affect quality of life and has a societal impact in terms of social withdrawal and impaired work performance.^7 8^ Each of these complaints could be defined as a distinct domain of tinnitus complaint. Currently, there are no objective assessment tools to measure the impact or severity of tinnitus. Assessment, diagnosis and evaluation are entirely reliant on self-report. Many multi-item tinnitus questionnaires have been published over recent decades and are often used as outcome measures to evaluate the effectiveness of interventions.^9^,^14^ In the context of clinical trials, an outcome is a measurement or observation used to assess the effectiveness or risk (such as side effects) of an intervention. For instance, outcomes collected in trials of tinnitus include tinnitus intrusiveness, tinnitus loudness, annoyance, intensity and distress.^15^ Therefore, it is perhaps unsurprising that across different trials of tinnitus interventions, the domain outcomes and measures used vary widely.

Currently, no treatment exists to eliminate tinnitus, but many interventions are being trialled. The psychological model of tinnitus proposed that tinnitus severity was underpinned by both the tinnitus characteristics and the psychological makeup of the patient, and that treatments for tinnitus may seek to address either the tinnitus percept or the psychological response to tinnitus.^16^ Therefore, different types of intervention for tinnitus with different mechanisms of action, seeking to address different aspects of tinnitus, require different outcomes to be measured to establish efficacy. Indeed, a systematic review identified eight broad classes of intervention for tinnitus and 35 primary domain outcomes spanning seven broad categories (tinnitus percept, impact of tinnitus, co-occurring complaints, quality of life, body structures and function, treatment-related outcomes and unclear or not specified). The most commonly reported outcome domains are tinnitus loudness (14% as a primary outcome and 7% as a secondary outcome) and tinnitus distress (7% as a primary outcome and 3% as a secondary outcome). The method of assessment for these outcome domains varied between studies, even when the same treatment outcome domain was being evaluated. For example, loudness was measured using either a numerical rating scale (8%), loudness matching (4%), minimum masking level (1%) and loudness discomfort level (1%). This lack of a standardised assessment can severely hinder the identification and interpretation of the relative merits of the various treatments that are currently on offer or interventions under investigation, and the most appropriate approaches for individual patients.^17^

A set of outcome domains and instruments that has been agreed on for a health condition is called a core outcome set.^18^ The purpose of a core outcome set is to define a minimum set of outcomes to be measured in every trial of a particular type of intervention in a specific area of health—in this case, electrical stimulation-based interventions for tinnitus. This does not necessitate that outcomes in a particular trial should be restricted to only those in the core outcome set. Rather, there is an expectation that the core outcome set will always be collected and reported, but additional outcomes can be measured. Defining what domains are important to measure will create the core domain set, which is the crucial first step in this process. It is important to identify outcome domains that are appropriate for the intervention strategy to have confidence that if a trial showed no effect, it did so because the intervention was not effective, and not because the outcomes measured were inappropriate for that particular intervention or population. A core outcome domain set developed from the perspectives of patients and professionals would address this. Perspectives of patients with the lived experience of the condition are important for understanding what matters to them. A recent systematic review demonstrated that the impact of interventions on patients’ lives is more likely to be represented in core domain sets that involve patients or their representatives.^19^ Instances have arisen where patients have identified outcomes as important that were previously overlooked^20^ or thought to be of little importance.^21 22^ Not every core domain set has been developed with patients’ input. A recent systematic review of patient participation in developing core domain sets found variability in study methods with no clear evidence on how to best promote patient recruitment.^23^ However, studies that involve patients in the study design are at least reasonably well placed to consider enablers and barriers to public participation during the study design phase.

The Core Outcome Measures in Tinnitus: International Delphi (COMiT’ID) study identified separate Core Domain Sets for sound-based, psychology-based and pharmacology-based interventions for tinnitus.^15^ At the time of the COMiT’ID study, electrophysiology interventions were excluded because available evidence was limited and dominated by a particular research group, while the other three interventions (sound therapy, psychological, pharmacological) encompassed broad international efforts in clinical research for tinnitus.^24^ In recent years, both the number of electrical stimulation interventions and studies evaluating those interventions have increased. Examples include increases in transcranial direct current stimulation (tDCS), transcranial alternating current stimulation (tACS), vagus nerve stimulation (VNS) and bimodal stimulation techniques, which combine non-invasive neural and acoustic stimulation.^25^ Due to these recent developments in electrophysiology, in particular increased efforts to develop treatment options and more clinical trials being reported, it became important to develop a core domain set for this type of intervention. Therefore, this study sought to build on previous work^15^ to develop a core domain set for electrical stimulation-based interventions for tinnitus.

## Methods

The full protocol for this study was published in advance.^26^

For the purposes of the COMiT: Electrical Stimulation (COMiT-ES) study, electrical stimulation-based interventions for tinnitus were defined as treatments that aim to improve tinnitus or its symptoms by electrical stimulation of the brain or other parts of the nervous system. This means that techniques that do not have a proposed mechanism that operates via the brain/nervous system, or that do not have improvements in tinnitus symptoms as their primary aim, were excluded. Furthermore, this core domain set does not cover devices such as cochlear implants or transcranial magnetic stimulation that rely on converting sound or magnetic energies into electrical pulses. Non-invasive brain stimulation methods such as tDCS, tACS and transcranial random noise stimulation (tRNS) and invasive methods such as VNS were included.

The aim of this study was to determine a core outcome domain set for electrical stimulation-based interventions for tinnitus. The objectives of this study were, therefore, to compare and integrate perspectives on outcome domains for electrical stimulation-based interventions for tinnitus through (1) a Delphi survey and (2) a consensus meeting with stakeholders. Key stakeholders were professionals working in the field of tinnitus (clinical practitioners, clinical researchers, commercial representatives and funders) and members of the public with lived experience of tinnitus.

### Patient and public involvement

All core outcome domains were reviewed and where necessary, reworded by patient representatives. All lay definitions of the core outcome domains presented were created in coproduction with patient representatives as part of the COMiT’ID study.^15^ For the purposes of this study, members of the NIHR Nottingham BRC Hearing Theme patient and public involvement (PPI) group reviewed all documents presented to participants, including patient information sheets and Delphi survey instructions, to ensure accessibility. When a new outcome was suggested in round 1 of the Delphi survey and included in round 2, this outcome and its definition were also reviewed by a patient representative.

### Research advisory group

A research advisory group was appointed to guide the project and aid in decision-making. The group comprises experts in the fields of tinnitus and core outcome set development (DAH and KF), a PPI and engagement manager and the study management team (BL, DH, LG and MS). The role of the research advisory group was to (1) support the development of the study protocol by providing feedback; (2) participate in online meetings to discuss progress on the Delphi study; (3) review study documentation, including information sheets for members of the public and professionals and intended advertisements; (4) participate in the piloting of the survey and (5) facilitate the online consensus meeting. None of the members of the research advisory group were allowed to vote on domains in the final consensus meeting.

### Eligibility criteria for the online surveys

Representatives from two stakeholder groups with relevant experience and/or interest in electronic stimulation for the management of chronic subjective tinnitus in adults were included. To be eligible for participation, participants had to be aged 18 or over and have a sufficient command of English to read, understand and complete questionnaires independently. Patients with lived experience of tinnitus (patient stakeholders) had to have experienced tinnitus for 3 months or more and had received a form of electrical stimulation for tinnitus (such as tDCS, tACS, tRNS or direct nerve stimulation) or would consider trying this type of treatment for their tinnitus. The professional stakeholder group included representatives from healthcare and clinical research. A targeted recruitment strategy was used to support the inclusion of an equal number of researchers and clinicians. These professionals were identified as representing the main professional categories in tinnitus research and clinical trials that would have representative homogeneous samples. Therefore, to be eligible, professional stakeholders had to meet one of the following four sets of criteria: (1) Clinically qualified and currently employed by a public or private institution that provides a tinnitus service to patients, experience of assessing, diagnosing or managing chronic subjective tinnitus and a working knowledge and/or clinical experience of electrical stimulation for tinnitus; (2) Academic qualification and currently employed by a research organisation, current or ‘recent past’ experience with studies that focus on questions of clinical efficacy of a tinnitus intervention in humans, with specific focus on interventions involving electrical stimulation (‘recent past’ was defined as having been an author on a relevant peer-reviewed journal publication in the past 3 years); (3) Currently employed by a company. that develops, manufactures or sells products that involve electrical stimulation that may be trialled for effectiveness in alleviating tinnitus and (4) Currently employed by an organisation that has funded tinnitus research projects addressing electrical stimulation-based interventions in the last 3 years. Journal editors were not included as a separate stakeholder group because it would not be possible to meet the minimum sample size requirement due to the small population size. However, given that the researchers included in the professional stakeholder group regularly act as journal editors and peer reviewers, this perspective was represented in the sample. During the introduction page of the online survey, both professionals and patients were asked to indicate informed consent and self-certify as being an ‘ex-pert’ in electrical stimulation-based interventions for tinnitus based on the above definitions.

### Panel size and justification

There is no agreed method to statistically calculate a sample size for Delphi surveys or for consensus meetings and no criteria against which a sample size choice can be judged.^27 28^ Some individual studies indicate that overall stakeholder groups of around 20 can provide results that are representative of the views of the wider stakeholder group.^27^ However, a key deciding factor is that the participant panel membership should adequately represent their corresponding stakeholder group. Another deciding factor is pragmatic where sample size is influenced by the aim of having a roughly equal number of participants in each stakeholder group.

### Recruitment methods

We used non-probabilistic purposive sampling to recruit to the Delphi survey, both from the UK and overseas. To ensure a representative sample of participants with a wide range of experience and perspectives, several recruitment strategies were employed for patient stakeholders via both clinical and non-clinical routes. Participants in the consensus meeting were recruited from among the participants in the Delphi survey.

### Preparatory work

The COMiT’ID study generated a long list of outcomes via a systematic review^24^ and narrative synthesis.^10^ The systematic review that yielded the original long list of outcomes prior to COMiT’ID included all trials of interventions of any type for tinnitus. The COMiT-ES team started off with the same original long list of outcomes, paying particular attention to those used to measure treatment-related change after electrical stimulation. The definitions in this list were coproduced by patient representatives.^29^ The COMiT’ID study long list of outcomes was modified for this study. Initially, the long list was inspected and reviewed by members of the Study Management Team (MS, DH and BL). Domains were removed if deemed irrelevant to electrical stimulation interventions. Exclusions were based on discussions of the Study Management Team and Advisory Group. Any duplications of outcomes were condensed, producing a final list of 64 domains. The names and definitions of the outcome domains, which had been reviewed by PPI as part of the COMiT’ID study, were not reviewed again. New materials, such as participant information sheets, were reviewed by members of the NIHR Nottingham BRC Hearing Theme PPI group. Members of the PPI group were asked to navigate the survey software and read the contents of the survey ahead of the launch of round 1 of the Delphi survey to test usability and face validity. Our PPI manager advised on the PPI strategy and facilitated contact with the PPI group before submission to the ethics committee. All outcome domains had plain language descriptions, generated for each outcome using an iterative process as part of the COMiT’ID study.^29^ The purpose of the plain language descriptions was to ensure that all domains were correctly interpreted by all stakeholders, including the patients, therefore, facilitating accurate and consistent understanding of domains across participant groups. To facilitate the presentation of the final long list, outcome domains were categorised into overarching domains. The final long list of categorised outcome domains was operationalised into questionnaire items (with the plain language description).

### The Delphi survey

**Figure 1:**
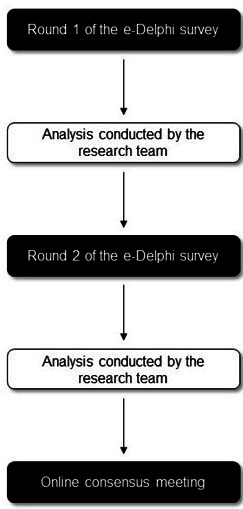
An overview of the Delphi process.

The Delphi survey comprised two sequential questionnaires or ‘rounds’ aiming to obtain a consensus on which outcome domains are most important in trials of electrical stimulation-based treatment for tinnitus, from the perspectives of professional and patient stakeholder groups. The Delphi survey was managed using DelphiManager, a bespoke online e-management system maintained by the COMET initiative.^30 31^ Each survey round contained a questionnaire that included the final long list of categorised outcome domains. An overview of the Delphi process is provided in figure 1.

Round 1: For each questionnaire item, participants were asked to think about the importance of a tinnitus outcome domain and indicate how important that domain is to measure when deciding whether an electrical stimulation-based tinnitus treatment is working. The outcome domains appeared in a randomised order to avoid order effects, while the outcomes within them were listed in alphabetical order to avoid potential weighting.^32^ Participants were asked to score each outcome domain using the Grading of Recommendations Assessment, Development and Evaluation (GRADE) scale of 1–9, where 1 represented least important and 9 represented most important.^33^ Selecting response options 1–3 was taken to indicate that the domain was considered ‘not important’, while 7–9 was taken to indicate that the domain was ‘critically important’ in deciding whether a tinnitus treatment is having its desired effect. Response options 4–6 were taken to indicate the outcome domain was considered ‘important but not critical’. If a participant felt that they did not understand a particular outcome, they were able to select ‘unable to score’. Participants had the option to suggest additional outcome domains for inclusion in round 2. These additional outcomes were reviewed and coded by two study management team members (BL and MS) to ensure they represented new outcomes. The distribution of the scores for each outcome domain was calculated for each stakeholder group within the Delphi survey.

Round 2: The purpose of round 2 was to enable participants to reflect on their scores considering the viewpoint of their own stakeholder group and the other stakeholder group in the Delphi survey. In the second round, participants were presented with the same list of outcome domains as in round 1, and any new outcomes identified by participants in round 1. Participants saw the same list of outcome domains with their own previous score, the anonymised distribution of scores across their stakeholder group and the anonymised distribution of scores from the other stakeholder groups from round 1. Results were presented graphically as well as numerically to improve visual appeal. Participants were asked to rescore the same list of outcome domains, considering this new information. To help give meaning to the GRADE scale, participants were reminded that any outcome domain would only be considered for inclusion in the core domain set if 70% of participants in each group select points 7–9 on the scale, and less than 15% select points 1–3.

### The online consensus meeting

Professionals and patients who had completed both rounds of the Delphi survey and registered an interest in participating in the consensus meeting were allocated places based on a first-come, first served basis. Eight participants—six professional stakeholders and two patient stakeholders—were recruited for participation in the consensus meeting. In advance of the meeting, participants were provided with an overview of the results of round 2, including a list of outcomes that met the inclusion definition according to ratings by both stakeholder groups and a list of outcomes that had been rated for inclusion by one stakeholder group, but not the other. They were asked to provide a top three of outcomes they felt were crucial to be included in the core domain set. The purpose of this was to steer both the participants’ thoughts and the conversation in the meeting towards what outcomes are critical. To minimise screen time during the web-based consensus meeting, participants were supplied with an overview of the aims of the day and a guidance document outlining the meeting activities. The meeting was conducted over Microsoft Teams and audio recorded and transcribed to facilitate reporting. The meeting comprised a plenary stage and breakout discussions in which the participants were allocated to groups along stakeholder lines. The meeting included anonymised voting on each outcome as either ‘in’ or ‘out’ which created pie charts and descriptive statistics which were displayed ‘live’ in the meeting. In line with the core domain sets resulting from the COMiT’ID study,^15^ the maximum number of outcomes in the outcome domain set was set a priori at seven (adverse effects and up to six further outcomes). Therefore, while the Delphi survey determined which outcomes were brought to the consensus meeting since there were more than seven outcomes, the consensus meeting participants were asked to reduce the number of outcomes. All Delphi survey participants were invited to agree or disagree with the decisions made at the consensus meeting to assess whether they enjoyed support among the wider sample.

### Consensus criteria

Consensus recommendations for the Delphi survey were made according to the following definition^18 34^: Include domain in core domain set: 70% or more of the participants in each stakeholder group score 7–9, and fewer than 15% score 1–3. Exclude outcome domains in the core domain set: 50% or fewer participants in each stakeholder group score 7–9. Consensus from the meeting was defined as 70% or more of the participants agreeing on including one or more outcome domains in the core domain set.

## Results

Using the planned recruitment strategy,^26^ 25 participants were recruited to the professional stakeholder group and 47 to the patient stakeholder group in round 1. In round 2, responses were received from 20 professional stakeholders and 36 patient stakeholders. Eight participants—six professional and two patient stakeholders—who took part in both Delphi survey rounds were recruited to the consensus meeting.

### Delphi survey

During round 1, participants were presented with 64 outcomes. For a complete list, see online supplemental materials. One outcome suggested by a participant in round 1 was included in round 2: Ability to differentiate tinnitus from external sounds, defined as the ability to tell your tinnitus apart from other sounds. This definition was generated for the new outcome by the study management team and reviewed by members of a local PPI group. All other outcomes suggested by participants in round 1 were either already covered by existing items on the Delphi survey or related to outcomes beyond the scope of this study. Round 2 comprised these same outcomes, as well as the additional outcome suggested by a participant in round 1: Ability to differentiate tinnitus from external sounds. Following the two-round Delphi, 8 outcome domains reached consensus criteria for inclusion, 22 met consensus criteria for exclusion and 10 were inconclusive due to meeting the consensus criteria for inclusion according to the ratings of one stakeholder group, but not the other. The remaining 25 outcome domains were inconclusive as they met neither the criteria for inclusion nor the criteria for exclusion. Table 1 details how each outcome was rated.

**Table 1:** Categorisation of outcomes according to inclusion criteria

Consensus to include	Inconclusive—included by one stakeholder group only	Inconclusive—no consensus for inclusion or exclusion	Consensus to exclude
Acceptance of tinnitus	Ability to ignore*	Ability to differentiate tinnitus from external sounds	Active myofascial trigger points
Concentration	Ability to relax*	Annoyance	Anger
Coping	Adverse reaction†	Anxiety	Behaviour
Tinnitus awareness	Depressive symptoms†	Brain structure	Bodily complaints
Tinnitus intrusiveness	Difficulties getting to sleep*	Change in sense of self	Catastrophising
Tinnitus loudness	Helplessness(lack of control)†	Conversations	Confusion
Tinnitus unpleasantness	Listening*	Distress from bodily sensations	Fat metabolism
Treatment satisfaction	Loss of peace*	Ill health	Fear
	Quality of sleep*	Impact on individual activities	Feeling tired
	Tinnitus pitch*	Impact on relationships	Gene expression
		Impact on social life	Joyful
		Impact on work	Lack of perceived support—nobody understanding experience
		Irritable	Loss of appetite
		Need for knowledge	Mood
		Negative thoughts/beliefs	Neck mobility
		Neural activity	Neck pain
		Neuroendocrine hormones	Nervous
		Oxidative stress	Seeking support
		Pain	Sexual difficulties
		Sense of control	Support from family and friends
		Suicidal thoughts	Tinnitus location
		Tinnitus quality	Upset
		Tinnitus-related thoughts	
		Withdrawal from treatment in the clinical trial	
		Worries/concerns	

* Met consensus criteria for inclusion by patient but not professional stakeholders.

† Met consensus criteria for inclusion by professional but not patient stakeholders.

### Consensus meeting

In advance of the consensus meeting, participants were provided the results of the Delphi survey as detailed in table 1. They were asked to select from the outcomes that met inclusion criteria and those that met inclusion criteria in one stakeholder group (the first two columns in table 1) three outcomes that they felt were most important to be included in the core domain set. All outcomes in the last two columns were excluded at this stage and were only provided to participants for context. Nine outcomes (quality of sleep, tinnitus unpleasantness, tinnitus pitch, loss of peace, difficulties getting to sleep, depressive symptoms, concentration and ability to relax) were not included by any participant in their ‘top 3’. At the outset of the consensus meeting, participants voted unanimously to exclude these outcomes and to limit discussions to the remaining nine (ability to ignore, acceptance of tinnitus, coping, helplessness (lack of control), listening, tinnitus awareness, tinnitus intrusiveness, tinnitus loudness and treatment satisfaction). However, following some further discussion, participants resolved to further include concentration and tinnitus unpleasantness in their discussions, despite none of them having included these in their ‘top 3’. Both these outcome domains had met the criteria for inclusion as rated by both stakeholder groups at the Delphi survey stage, which the participants felt obliged them to consider in their discussions.

Small group and whole group discussions led to various decisions within the meeting. Acceptance of tinnitus was excluded by participants as this outcome domain was considered more relevant to other types of treatment, in particular psychological interventions for tinnitus. Coping was excluded for the same reason. It is worth noting that participants discussed acceptance of tinnitus, coping and ability to ignore together, identifying them as ‘triplets’. Of the three, the ability to ignore was considered the most relevant to electrical stimulation-based interventions for tinnitus, with a beneficial effect of such an intervention potentially facilitating the ability to ignore the tinnitus percept. Listening was excluded with participants again reasoning that this outcome would be of interest to studies aimed at answering questions relating to tinnitus and hearing or listening ability, but would not be a core outcome, critical to measure in trials of electrical stimulation for tinnitus. Moreover, the participants argued that a mechanism for electrical stimulation interventions to affect listening appeared to be lacking. Tinnitus awareness, tinnitus loudness, tinnitus unpleasantness and tinnitus intrusiveness were discussed together and compared by participants. Participants felt that both tinnitus awareness and tinnitus unpleasantness were covered by tinnitus intrusiveness. Furthermore, they judged tinnitus intrusiveness to be a more meaningful outcome to assess whether an electrical stimulation intervention for tinnitus was successful than tinnitus loudness, as the two may not be strongly correlated.

Following the synthesis of discussion points, six outcomes were put forward for a vote on inclusion in the final core domain set: ability to ignore, concentration, treatment satisfaction, helplessness (lack of control), tinnitus intrusiveness and tinnitus loudness. Only tinnitus loudness did not reach consensus level agreement for inclusion. The core outcome domain set recommendations resulting from the consensus meeting, including the outcome domains and their lay definitions can be found in table 2.

**Table 2:** COMiT-ES recommendation for a core outcome domain set for electrical stimulation-based interventions for tinnitus

Outcome domain	Definition
Ability to ignore	Ability to continue as normal as if tinnitus were not there
Concentration	The ability to continue as if tinnitus were not there
Treatment satisfaction	How the treatment meets your expectations or how pleased you are after receiving the treatment
Helplessness (lack of control)	Feeling despair about being unable to control or manage tinnitus
Tinnitus intrusiveness	The extent to which tinnitus invades your life, stresses you in daily situations and prevents you from doing things you want to do. The unacceptable and unwelcome interference of internal head and body noise heard only by the individual. Being acutely aware of the sounds of tinnitus, feeling that it is invading your life or your personal space, changing your thoughts or actions and negatively impacting your life

COMiT-ESCore Outcome Measures in Tinnitus: Electrical Stimulation

Table 3 provides a comparison to previously established core outcome domain sets for psychological, pharmacological and sound-based interventions for tinnitus.

**Table 3:** Comparison of current core outcome domain set with previously established core outcome domain sets for tinnitus intervention types

Sound	Psychology	Pharmacology	Electrical stimulation
Tinnitus intrusiveness	Tinnitus intrusiveness	Tinnitus intrusiveness	Tinnitus intrusiveness
Ability to ignore	Tinnitus acceptance	Tinnitus loudness	Ability to ignore
Concentration	Mood		Concentration
Quality of sleep	Negative thoughts and beliefs		Helplessness (lack of control)
Sense of control	Sense of control		Treatment satisfaction

22 participants from the Delphi survey who were not included in the consensus meeting responded to a request to indicate how satisfied they were with the core outcome domain set (table 4), of whom 19 (86%) said they were either somewhat or very satisfied with the final set. Only two participants indicated they were somewhat dissatisfied, and no one was very dissatisfied. Since this feedback was collected anonymously to encourage candour, it is not possible to determine which stakeholder group the participants who were somewhat dissatisfied belonged to. However, both expressed surprise that tinnitus loudness had not been included.

**Table 4:** Results of feedback from participants who did not take part in the consensus meeting on the final core outcome domain set

How satisfied are you with the choice of outcome domains included in the final core outcome domain set?	(n=22)
I am very satisfied with the choice of included outcome domains	63.6%
I am somewhat satisfied with the choice of included outcome domains	22.7%
I am neither satisfied nor dissatisfied	4.5%
I am somewhat dissatisfied with the included outcome domains	9.1%
I am very dissatisfied with the included outcome domains	0%

## Discussion

This study established a core outcome domain set for the evaluation of electrical stimulation-based interventions for tinnitus. The resulting core outcome domain set will act as a minimum standard for reporting in future clinical trials of electrical stimulation interventions for tinnitus. To ensure this core outcome domain set reflects the priorities of all relevant stakeholders, this study drew on the expertise of patients, clinicians, researchers and other stakeholders.

Several challenges presented themselves while this study was being conducted. The COVID-19 pandemic necessitated that all data collection took place online, including the consensus meeting. This was particularly challenging to the recruitment of patient stakeholders to the meeting. Age is a major risk factor for tinnitus, and tinnitus frequently co-occurs with hearing loss and hyperacusis. Many of the patient stakeholders declined the invitation to take part in the consensus meeting, citing a lack of ability or confidence in the use of software, hearing loss, hyperacusis or a combination of these factors as reasons to decline. As a result, comparatively few patient stakeholders took part in the consensus meeting. All possible efforts to remove barriers to taking part in the consensus meeting were taken, primarily following the approaches used in the CROSSSD (Core Rehabilitation Outcome Set for Single-Sided Deafness) study.^35^

The core outcome domain set for electrical stimulation-based interventions for tinnitus complements the existing psychological, pharmacological and sound-based interventions for tinnitus. The current core outcome domain set shares tinnitus intrusiveness with all of those previously established and further shares ability to ignore and concentration with the core outcome domain set for sound therapy for tinnitus. The authors interpret this as an indication that, while appropriately intervention-specific, this core outcome domain set reflects the priorities of people with tinnitus and of the clinical and research community. Participants who took part in the Delphi survey but not the consensus meeting were largely either very satisfied or somewhat satisfied with the final Core Outcome Domain Set. This indicates that support for the Core Outcome Domain Set reaches beyond the participants in the consensus meeting.

The recommended core outcome domain set reported here is intended as a minimum reporting standard for future clinical trials of electrical stimulation-based interventions for tinnitus in adults. Consistency in the reporting of outcomes in clinical trials will facilitate the pooling of data from such trials in meta-analyses, in turn facilitating the use of data from such trials in evidence-based medicine. While this minimum reporting standard should always be measured in clinical trials of electrical stimulation-based interventions for tinnitus, they are not intended to limit outcome measurement. Investigators should not feel restricted in any way to add outcome measurements that may be of interest to the study-specific aims or hypotheses in their trial design.

While no instruments have been defined with which the authors propose the outcomes in this set should be measured, the Tinnitus Functional Index, already widely used in interventional tinnitus studies, could be used to measure all outcomes in this set, save for treatment satisfaction, which could be measured using the Short Assessment of Patient Satisfaction or another appropriate validated instrument. While in the past, outcome reporting in clinical trials of electrical stimulation-based interventions for tinnitus has been inconsistent, widespread adoption of this core outcome domain set could bring a consistency in data collection and reporting to future trials that would facilitate data pooling and thereby accelerate the building of a robust empirical evidence base for, or against, interventions of this type for tinnitus.

## supplementary material

10.1136/bmjopen-2023-079769 online supplemental file 1

## Data Availability

Data are available on reasonable request.
