# Supplementary material for: Establishing a core domain set for early-phase clinical trials of electrical stimulation interventions for tinnitus in adults: an online Delphi study
Source: BMJ Open. 2025 Mar 4;15(3):e079769. doi: 10.1136/bmjopen-2023-079769 (PMC11881199; doi:10.1136/bmjopen-2023-079769)
Supplement: Supplementary file 2 [file bmjopen-2023-079769.reviewer_comments.pdf]

PEER REVIEW HISTORY

BMJ Open publishes all reviews undertaken for accepted manuscripts. Reviewers are asked to complete a checklist review form and are provided with free text boxes to elaborate on their assessment. These free text comments are reproduced below.

ARTICLE DETAILS

Title (Provisional)

Establishing a Core Domain Set for early-phase clinical trials of electrical stimulation interventions for tinnitus in adults: an online Delphi study

Authors

Labree, Bas; Hoare, Derek; Fackrell, Kathryn; Hall, Deborah A; Gascoyne, Lauren; Sereda, Magdalena

---

VERSION 1 - REVIEW

**Reviewer**	**1**
**Name**	**Yu, Huiqian**
**Affiliation**	**Fudan University Eye Ear Nose and Throat Hospital**
**Date**	**27-Oct-2023**
**COI**	**No competing interests**

I think that this is a meaningful study with regard to prospective nature and methodology. There are several comments to improve the manuscript.

Major comments
1. Will different electrical stimulation methods affect the outcomes? In this study, electrical stimulation interventions for tinnitus had a variety of stimulation methods, including non-invasive brain stimulation methods and invasive brain stimulation methods. This difference may have influenced participants' decisions about the importance of the Core Domain Set.

2. I think it is necessary to add the concept of the Delphi study and recent developments in electrical stimulation interventions so that readers can better understand your study.
3. Prior to the consensus meeting, participants were asked to select the "top 3" outcomes. Would the outcome domains be better assessed if they were asked to sequence the first seven outcomes that they considered important?
4. Acceptance of tinnitus, coping and ability to ignore have been discussed together and identified as "triplets". What is the difference between "triplets"? And why ability to ignore is more important than the other two outcomes?
5. Tinnitus awareness, tinnitus loudness, tinnitus unpleasantness and tinnitus intrusiveness were discussed together. But how to define tinnitus intrusiveness as a more meaningful outcome?
6. Two of the 22 participants who did not participate in the consensus meeting expressed somewhat dissatisfaction to the final set. Which stakeholder group did they come from? And what were the reasons for their dissatisfaction?

**Reviewer**	**2**
**Name**	**Tyler, Richard**

Affiliation The University of Iowa, Otorhinolaryngology

Date 24-Sep-2024

COI No competing interests.

Submitted on behalf of reviewer.

Reviewers comments:

Here is my review

Generally, I think it would be very helpful to distinguish the Tinnitus from the Reactions to the tinntus…..Please cite the Psychological Model, proposed by Tyler, Aran and Dauman (1992), suggested that the overall annoyance of the tinnitus was a result of the 1) tinnitus characteristics and the 2) psychological make up of each individual patient. Several parts of the brain will be involved in the representation of the tinnitus and of the reactions to the tinnitus. Treatments can be focused on reducing the tinnitus (e.g. pills) or on reducing the reactions to the tinnitus (eg. Counseling)

Tyler, R. S., Aran, J-M., & Dauman, R. (1992). Recent advances in tinnitus. Am J Audiol, 1(4): 36-44.

Also see

Dauman, R. & Tyler, R. S. (1992). Some considerations on the classification of tinnitus. In: J-M. Aran & R. Dauman (Eds.), Tinnitus 91 - Proceedings of the Fourth International Tinnitus Seminar (225-229). Amsterdam: Kugler Publications.

This classification is further elaborated on in the review chapter by Tyler (2006) with detailed review of the literature. You should cite these;

Tyler, R. S. (2006). Neurophysiological Models, Psychological Models, and Treatments for Tinnitus. Chapter 1 (p1-22). In R.S. Tyler (Ed.), Tinnitus Treatment: Clinical Protocols (1-22). New York: Thieme.

Quality of Life scales typically do not appreciate the importance of hearing (and tinnitus). I question their validity. This could also be mentioned. Please cite ,

Tyler R, Perreau A, Mohr AM, Ji H, Mancini PC (2019). An Exploratory Step Toward Measuring the 'Meaning of Life' in Patients with Tinnitus and in Cochlear Implant Users. J Am Acad Audiol. 2019 Sep 27. doi: 10.3766/jaaa.19022.

**Reviewer**	**3**
**Name**	**Keane, David**
**Affiliation**	**Leeds Teaching Hospitals NHS Trust, Renal medicine**
**Date**	**08-Oct-2024**
**COI**	**N/a**

This online Delphi study recruited individuals with experience of tinnitus as healthcare users (n=25) and researchers/healthcare professionals (n=47) to establish consensus on a core domain set for early phase clinical trials of electrical stimulation therapy for tinnitus. Two rounds of a survey was followed up by an online meeting (8 people) to finalise domains.

1) I think the introduction could flow better than it does presently. The first paragraph feels a little disjointed as it moves between discussion of the clinical condition, use of assessments in the management of tinnitus and into outcomes for trials. Could the paragraph starting "Currently, no treatment exists" directly follow the description of the clinical condition to clearly state why this work is needed?

2) A very clear description earlier in the introduction of how a core domain set fits into a core outcome would be helpful.

3) I think the description of the methodological aspects of core outcome set development is too long and not appropriate for the introduction and should largely be considered in the discussion or removed.

4) The final paragraph on page 5 is a nice statement of need for this study. The following paragraph is methodology and should be moved out of the introduction.

5) First paragraph of results the term "patient" is used while elsewhere it is "healthcare user" – suggest using one term throughout.

6) I think the line "The definitions in this list were subject to Patient and Public Involvement (PPI) from the stakeholder groups in this study" could be better phrased to explain the role of the PPI group.

7) The discussion is very short. I think this could be expanded by discussing the clinical implications of the domains chosen and bringing the narrative back to the aims of the study in the introduction.

VERSION 1 - AUTHOR RESPONSE

Reviewer: 1

Dr. Huiqian Yu, Fudan University Eye Ear Nose and Throat Hospital

Comments to the Author:

I think that this is a meaningful study with regard to prospective nature and methodology. There are several comments to improve the manuscript.

Major comments
1. Will different electrical stimulation methods affect the outcomes? In this study, electrical stimulation interventions for tinnitus had a variety of stimulation methods, including non-invasive brain stimulation methods and invasive brain stimulation methods. This difference may have influenced participants' decisions about the importance of the Core Domain Set.

The definition of electrical stimulation for tinnitus, for the purpose of this Core Outcome Domain Set was carefully formulated to ensure it was applicable widely enough to be practical, but narrow enough to ensure all stimulation techniques that fall under it would be comparable in, for instance, mechanism of action. For context, cochlear implants were explicitly excluded, as they are primarily an intervention for hearing loss, not tinnitus. Transcranial Magnetic Stimulation (TMS) and Focused Ultrasound Stimulation were excluded on the basis that their mechanisms of action were too different from electrical stimulation methods. Whilst participants might only have direct experience of one type of electrical stimulation, their views would still be relevant to all types included in this definition.

2.I think it is necessary to add the concept of the Delphi study and recent developments in electrical stimulation interventions so that readers can better understand your study.

Recent developments in electrical stimulation-based interventions for tinnitus have been elaborated upon in the Introduction. The application of Delphi methodology is described in detail in the Methods.

3.Prior to the consensus meeting, participants were asked to select the “top 3” outcomes. Would the outcome domains be better assessed if they were asked to sequence the first seven outcomes that they considered important?

Prior experience with Delphi methodology for Core Outcome Domain establishment has taught us that participants tend to respond in favour of inclusion. This makes it difficult to find consensus on a limited number of outcome domains. Asking participants to effectively formulate their own Core Outcome Domain by asking them for a top 7 (would have had to be a top 6, as one outcome domain is always *adverse effects*) would have left little room for discussion and consensus building. Asking participants to choose more restrictively forced them to reflect very carefully on what they considered “core” outcomes. This technique was previously employed in the CROSSSED study (https://www.comet-initiative.org/studies/details/1084). It is worth noting that, the Top 3s served as a starting point for the consensus meeting. The participants had the opportunity to discuss all outcome domains, but chose to limit themselves to the Top 3s, as well as outcome domains which had met the consensus criteria for inclusion in the Delphi survey.

4. Acceptance of tinnitus, coping and ability to ignore have been discussed together and identified as "triplets". What is the difference between "triplets"? And why ability to ignore is more important than the other two outcomes?

Participants grouped these outcome domains together during their discussions. Each of these outcome domains had its own lay definition, produced by the PPI efforts of the Commit-ES study. The conclusion that *ability to ignore* should be included and the others not, lay with the stakeholders. The stakeholders made decisions to include or exclude outcomes independently, based on their respective expertise. The authors did not influence these decisions and it would not be appropriate for them to attempt to justify the inclusion or exclusion of any given outcome domain by the stakeholders.

5. Tinnitus awareness, tinnitus loudness, tinnitus unpleasantness and tinnitus intrusiveness were discussed together. But how to define tinnitus intrusiveness as a more meaningful outcome?

Participants grouped these outcome domains together during their discussions. Each of these outcome domains had its own lay definition, produced by the PPI efforts of the Commit-ES study. The purpose of this study was not to redefine outcomes, so addressing this issue is beyond its scope.

6. Two of the 22 participants who did not participate in the consensus meeting expressed somewhat dissatisfaction to the final set. Which stakeholder group did they come from? And what were the reasons for their dissatisfaction?

Feedback on the Core Outcomes Domain Set was recorded anonymously to encourage candour, so it isn’t possible to determine which stakeholder group these participants belonged to. This has been clarified in the manuscript. Both remarked they were surprised *tinnitus loudness* had not been included.

Reviewer 2:

Prof. Richard   Tyler, The University of Iowa

Comments to the Author:

Submitted on behalf of reviewer.
Reviewers comments:
Here is my review

Generally, I think it would be very helpful to distinguish the Tinnitus from the Reactions to the tinntus…..Please cite the Psychological Model, proposed by Tyler, Aran and Dauman (1992), suggested that the overall annoyance of the tinnitus was a result of the 1) tinnitus characteristics and the 2) psychological make up of each individual patient. Several parts of the brain will be involved in the representation of the tinnitus and of the reactions to the tinnitus. Treatments can be focused on reducing the tinnitus (e.g. pills) or on reducing the reactions to the tinnitus (eg. Counseling)

Tyler, R. S., Aran, J-M., & Dauman, R. (1992). Recent advances in tinnitus. Am J Audiol, 1(4): 36-44.

The Introduction has been edited to include the Psychological Model.

Also see

Dauman, R. & Tyler, R. S. (1992). Some considerations on the classification of tinnitus. In: J-M. Aran & R. Dauman (Eds.), Tinnitus 91 - Proceedings of the Fourth International Tinnitus Seminar (225-229). Amsterdam: Kugler Publications.

This classification is further elaborated on in the review chapter by Tyler (2006) with detailed review of the literature. You should cite these;

Tyler, R. S. (2006). Neurophysiological Models, Psychological Models, and Treatments for Tinnitus. Chapter 1 (p1-22). In R.S. Tyler (Ed.),   Tinnitus Treatment: Clinical Protocols (1-22). New York: Thieme.

Quality of Life scales typically do not appreciate the importance of hearing (and tinnitus). I question their validity. This could also be mentioned. Please cite ,

Tyler R, Perreau A, Mohr AM, Ji H, Mancini PC (2019). An Exploratory Step Toward Measuring the 'Meaning of Life' in Patients with Tinnitus and in Cochlear Implant Users. J Am Acad Audiol. 2019 Sep 27. doi: 10.3766/jaaa.19022.

Where appropriate, the suggested works have been referenced. However, some of the suggested publications are outdated, beyond the scope of this manuscript, or both.

Dr. David Keane, Leeds Teaching Hospitals NHS Trust

Comments to the Author:

This online Delphi study recruited individuals with experience of tinnitus as healthcare users (n=25) and researchers/healthcare professionals (n=47) to establish consensus on a core domain set for early phase clinical trials of electrical stimulation therapy for tinnitus. Two rounds of a survey was followed up by an online meeting (8 people) to finalise domains.

1) I think the introduction could flow better than it does presently. The first paragraph feels a little disjointed as it moves between discussion of the clinical condition, use of assessments in the management of tinnitus and into outcomes for trials. Could the paragraph starting "Currently, no treatment exists" directly follow the description of the clinical condition to clearly state why this work is needed?

The Introduction has been edited to improve flow. The paragraph starting "Currently, no treatment exists…" has been moved to directly follow the paragraph with the description of tinnitus.

2) A very clear description earlier in the introduction of how a core domain set fits into a core outcome would be helpful.

A core domain set does not fit into a core outcome. A Core Outcome Set comprises all outcomes that should be measured in every trial of a particular (class of) intervention for a given condition, as well as the respective instruments with which they should be measured. A Core Outcome Domain Set does not define these instruments. The Introduction has been restructured to clarify this.

3) I think the description of the methodological aspects of core outcome set development is too long and not appropriate for the introduction and should largely be considered in the discussion or removed.

The description in the introduction has been shortened, with some elements moved to the Methods section.

4) The final paragraph on page 5 is a nice statement of need for this study. The following paragraph is methodology and should be moved out of the introduction.

The paragraph in question has been removed from the Introduction.

5) First paragraph of results the term “patient” is used while elsewhere it is “healthcare user” – suggest using one term throughout.

The term “healthcare user” has been replaced by “patient” throughout the manuscript.

6) I think the line “The definitions in this list were subject to Patient and Public Involvement (PPI) from the stakeholder groups in this study” could be better phrased to explain the role of the PPI group.

The exact nature of the PPI contribution to the lay definitions has been described in greater detail within the Patient and Public Involvement section.

7) The discussion is very short. I think this could be expanded by discussing the clinical implications of the domains chosen and bringing the narrative back to the aims of the study in the introduction.

The Discussion has been extended to reflect more extensively on the implications of the results.

VERSION 2 - REVIEW

**Reviewer**	**3**
**Name**	**Keane, David**
**Affiliation**	**Leeds Teaching Hospitals NHS Trust, Renal medicine**
**Date**	**13-Jan-2025**
**COI**	

Thank you for the response. No further comments.
